# Development of Spray-Dried Mannitol–Pregelatinized Rice Starch Using SeDeM-Based Approach for Direct Compressible Cetirizine Dihydrochloride Tablets

**DOI:** 10.3390/pharmaceutics17111409

**Published:** 2025-10-30

**Authors:** Phennapha Saokham, Ruttiros Khonkarn, Pratchaya Tipduangta, Pattaraporn Panraksa, Karnkamol Trisopon

**Affiliations:** Department of Pharmaceutical Sciences, Faculty of Pharmacy, Chiang Mai University, Chiang Mai 50200, Thailand; phennapha.s@cmu.ac.th (P.S.); ruttiros.khonkarn@cmu.ac.th (R.K.); pratchaya.t@cmu.ac.th (P.T.); pattaraporn.pan@cmu.ac.th (P.P.)

**Keywords:** co-processed excipients, direct compression, spray drying, SeDeM expert system, cetirizine dihydrochloride

## Abstract

**Background/Objectives**: Direct compression offers a cost-effective route for tablet manufacturing but is often limited by poor powder flow and compressibility. This study reported the development of a co-processed excipient comprising 98% mannitol and 2% pregelatinized rice starch (PRS) using spray drying with ammonium bicarbonate as a pore-forming agent. **Methods**: This optimized excipient demonstrated balanced powder flow and enhanced compressibility suitable for direct compression applications. The SeDeM expert system guided the optimization process by evaluating raw and spray-dried components. PRS exhibited excellent flowability that decreased after spray drying but displayed significantly enhanced compressibility, whereas mannitol maintained superior flow but continued to show limited compressibility post-drying. Scanning electron microscopy, differential scanning calorimetry, Fourier-transform infrared spectroscopy, and X-ray powder diffraction confirmed the absence of chemical interactions and unchanged wettability during co-processing. **Results**: The resulting excipient combined the favorable flow characteristics of mannitol with the improved compressibility of PRS, rendering it suitable for direct compression. Cetirizine dihydrochloride (CET) tablets were formulated via exponential curve fitting within the SeDeM framework, yielding an optimal CET-to-excipient ratio of 13:87. The tablets met all pharmacopeial physicochemical requirements, including uniform mass, adequate tensile strength, rapid disintegration, and dissolution profiles comparable to a reference product, with dissimilarity (f_1_ = 4.28) and similarity (f_2_ = 64.03) factors within regulatory acceptance limits. **Conclusions**: These findings represented the first application of SeDeM methodology to a co-processed mannitol–pregelatinized rice starch system, enabling predictive optimization of powder flow and compressibility in direct compression formulations.

## 1. Introduction

Tablets are among the most widely used dosage forms, favored for their precision in dosing, portability, and generally offering cost-effective production [1]. Typically tablets comprise one or more active pharmaceutical ingredients (APIs) along with excipients, and they are manufactured by compressing powdered particles [2]. The three main manufacturing methods include wet granulation, dry granulation, and direct compression. Among these, direct compression is the simplest and most cost-effective approach [3]. This technique reduces the production complexity by minimizing the number of steps and equipment while enhancing tablet stability by avoiding exposure to water and heat during manufacturing [1]. However, the effectiveness of direct compression depends on the selection of excipients with both adequate flowability and compressibility [2]. Unfortunately, direct compression is often limited by the inherent trade-off between powder flowability and compressibility. Many APIs and excipients fail to exhibit these properties simultaneously; some may have excellent compressibility but poor flowability, whereas others may show the opposite [3]. To overcome these challenges, many researchers have focused on developing direct compression (DC) excipients designed to optimize these critical properties while ensuring uniform particle size and compatibility with other formulation components [4].

A prominent strategy for improving these excipients is co-processing, where two or more excipients are physically combined without altering their chemical structure [2]. Co-processing enables physical integration of multiple excipients to synergistically improve tablet properties without altering individual chemical structures, offering advantages over simple blends in mechanical strength and performance. Compared to simple physical mixtures, co-processed excipients offer improved mechanical properties, faster disintegration, and enhanced performance in tablet formulations. By leveraging the distinct mechanical behaviors of excipients—such as plastic deformation and brittle fracture—co-processing modifies their physicochemical properties to improve compressibility and flowability [5,6]. For instance, commercial examples include Cellactose^®^, which combines lactose and cellulose to achieve optimal compression characteristics, and StarLac^®^, which blends lactose with corn starch [7].

In this study, mannitol was selected as the primary excipient in the co-processed system due to its excellent physicochemical properties, including high aqueous solubility, excellent flowability, pleasant mouthfeel, and low hygroscopicity, which make it highly suitable for direct compression tablet formulations [8]. Additionally, its compatibility with a wide range of excipients and low tendency for chemical interaction supports its selection as the dominant component of formulation. However, crystalline mannitol is known to exhibit relatively poor compressibility and brittle fracture behavior during compaction, often resulting in tablets with weak mechanical strength [9]. To overcome these limitations, co-processing mannitol with an optimum proportion of an excipient exhibiting plastic deformation behavior was employed. This brittle-plastic combination enhances the compactibility, mechanical strength, and overall performance of the co-processed excipient in direct compression applications [6].

Among native starches, rice starch (*Oryza sativa*), is particularly noteworthy due to its distinct composition of amylose and amylopectin [10]. The ratio of these components plays a crucial role in disintegration, cohesion, and compressibility [11]. Specifically, rice starch, containing approximately 20% amylose, is soluble in hot water while insoluble in cold water and ethanol [12]. Although rice starch can enhance compressibility, its small particle size (2–7 μm) poses limited flowability challenges. Structural modifications, such as gelatinization through thermal or alkaline treatments, can enhance the functional properties of rice starch by increasing its viscosity and modifying its structural characteristics, making it more suitable for binding and disintegration in solid dosage forms [13].

Porous microspheres, used in various applications including drug delivery, can also benefit from processes like spray drying. The ability to create internal and external pores allows for improved flow and drug release [14]. Key parameters influencing microsphere properties include diameter, pore volume, and pore architecture. The incorporation of porogens during processing enhances these properties by creating void spaces that improve flowability, compressibility, and drug delivery efficiency [15].

To assess the suitability of materials for direct compression, the SeDeM (Sediment Delivery Model) expert system is a valuable tool [16]. This system approach evaluates critical parameters such as flowability and compressibility, providing a comprehensive analysis of material properties [17]. By converting these values into radius metrics, the SeDeM model aids in assessing the suitability of materials for direct compression.

In this study, the SeDeM system was applied to characterize and optimize co-processed materials between pregelatinized rice starch and mannitol prepared via spray drying using ammonium bicarbonate as a porogen. The objective was to develop a direct compression diluent tailored for immediate-release tablet formulations, using cetirizine dihydrochloride (CET) as a model drug with poor flow properties [18].

## 2. Materials and Methods

### 2.1. Materials

Rice starch (food grade) was sourced from the Thai Flour Industry Co., Ltd., Bangkok, Thailand. Mannitol was procured from Kemaus, Cherrybrook, NSW, Australia. Ammonium bicarbonate (NH_4_HCO_3_) was acquired from Loba Chemie, Mumbai, India. Cetirizine dihydrochloride (CET) was purchased from Ambeed, Inc., Buffalo Grove, IL, USA. Sodium starch glycolate was generously supplied by Onimax Co., Ltd., Bangkok, Thailand, while sodium stearyl fumarate (Lot WUBC82, food grade) and talc were obtained from MySkinRecipes, Bangkok, Thailand. Zyrtec^®^, cetirizine dihydrochloride film-coated tablets, Lot 372767 Mfd. 2 June 2023 Exp. 31 May 2028 as a reference product were manufactured by UCB Farchim SA, Bulle, Switzerland. HPLC-grade methanol was obtained from RCI Labscan, Bangkok, Thailand, and deionized (DI) water was produced using the Optima 10 system from ELGA Labwater, High Wycombe, UK. Unless otherwise specified, all chemicals were of analytical reagent (AR) grade.

### 2.2. Preparation of Pregelatinized Rice Starch (PRS)

Rice starch was dispersed in water at a concentration of 0.4 g/mL. The suspension gradually heated to 60 ± 2 °C with continuous stirring. Heating ceased once a portion of the starch commenced pregelatinizing at the surface, at which point the temperature stabilized. The resulting pregelatinized rice starch solution was transferred into an aluminum tray and subjected to drying in an oven at 50 ± 5 °C overnight. Post-drying, the pregelatinized rice starch was ground using an oscillating granulator equipped with a #20 mesh screen. The ground pregelatinized rice starch was subsequently passed through a #80 mesh sieve before being stored in a desiccator.

### 2.3. Determination of Degree of Gelatinization

The thermal behavior of PRS and rice starch was analyzed using differential scanning calorimeter (DSC-1, Mettler-toledo, Greifensee, Switzerland) to determine the degree of gelatinization according to Yusop et al. [19]. Samples were weighed into aluminum pans and assessed over a temperature range of 25–120 °C at a heating rate of 10 °C/min. Before hermetically sealing, 10 μL of deionized water was added. Normalized enthalpy (ΔH) was calculated, and the degree of gelatinization was derived using Equation (1):

(1) $$\mathrm{Degree}\;\mathrm{of}\;\mathrm{gelatinization}\;=\;100\;-\;\left(\frac{{∆H}_{\mathrm{PRS}}}{{∆H}_{\mathrm{Rice}\;\mathrm{starch}}}\;\times \;100\right)$$

where ∆H_PRS_ and ∆H_Rice starch_ are the normalized gelatinization enthalpy of PRS and rice starch, respectively.

### 2.4. Preparation of Spray Dried Excipients

Porous excipient particles were fabricated using a Mini Spray Dryer B-290 (Buchi, Flawil, Switzerland) equipped with a 0.7 mm nozzle. A spray suspension, consisting of mannitol, PRS, or a combination of both in distilled water, was prepared by continuous stirring at ambient temperature (approximately 25–30 °C) for 30 min. For porous particle fabrication, ammonium bicarbonate was added, and the mixture was stirred for an additional 10 min, covered with aluminum foil. Based on preliminary experiments, the optimal ratio of excipient to ammonium bicarbonate was determined to be 5:1, corresponding to 2.5% *w*/*w* excipient and 0.5% *w*/*w* ammonium bicarbonate. The spray drying process was performed under co-current air flow conditions at a rate of 473 L per hour, with an aspirator rate of 100%, a pump setting as 4 mL/min, and an inlet temperature of 160 °C. The final product was collected from the collector and stored in a polyethylene bag inside a desiccator.

### 2.5. Powder Characterization Using the SeDeM Expert System

The powder characterization of mannitol, rice starch, their spray-dried products, and cetirizine dihydrochloride was conducted using the SeDeM expert system as described by Suñé-Negre and colleagues [16]. Twelve parameters—bulk density (D_a_), tapped density (D_c_), inter-particle porosity (Ie), Carr index (IC), cohesion index (Icd), Hausner ratio (IH), angle of repose (α), powder flow time (t″), loss on drying (%HR), hygroscopicity (%H), percentage of particles < 45 μm (%Pf), and homogeneity index (Iθ)—were measured following European Pharmacopeia. Each measured value (v) was converted to a radius metric (0–10) using established SeDeM conversion equations validated by Aguilar-Díaz and coworkers [20] (Table 1) and plotted as a 12-vertex polygon. Incidence factors for Dimension, Compressibility, Flowability, Lubricity/Stability, and Lubricity/Dosage were calculated as the means of their respective radii. Index Parameter (IP), Parameter Profile Index (IPP), and Index of Good Compressibility (IGC) were computed using Equations (11)–(13), with acceptance thresholds of 0.5 for IP and 5.0 for both IPP and IGC.

These twelve parameters, which are categorized into five factors, were determined through both experimental and quantitative mathematical methods of powder characterization, as follows:

- Dimension: Bulk density (D_a_) and tapped density (D_c_) were determined in accordance with the European Pharmacopeia guidelines (2.9.34). A measured mass (M) of the powder was placed into a 100 mL graduated cylinder, and the bulk volume (V_a_) was recorded. Bulk density was then calculated using Equation (2). The cylinder containing the powder was tapped continuously using a Jolting volumeter (Stav 2003, Erweka, Langen, Germany) for 1250 taps, after which the tapped volume (V_c_) was recorded. Tapped density was subsequently calculated from the mass (M) and tapped volume (V_c_) using Equation (3).

(2) $$D_{a}=\frac{M}{V_{a}}$$

(3) $$D_{c}\;=\frac{M}{V_{c}}$$

- Compressibility: Inter-particle porosity (Ie) and Carr Index (IC) were calculated from the bulk and tapped densities using Equations (4) and (5), respectively.

(4) $$\mathrm{Ie}=\frac{D_{c}\;-\;D_{a}}{D_{c}\;\times \;D_{a}}$$

(5) $$\mathrm{IC}=\frac{100\;\times \;(D_{c}\;-\;D_{a})}{D_{c}}$$

- The Cohesion Index (Icd) was employed to determine the compressive stress of a tablet. For this assessment, 500 mg of powder was compressed into an 11 mm flat-face tablet using a hydraulic tablet press (C, Carver, Wabash, IN, USA) under a compression force of 0.1 MPa. The tablet’s crushing strength (N) was then measured using a tablet hardness tester (PTB-311 PharmaTest, Hainburg, Germany) and divided by the applied compression pressure.
- Flowability: The Hausner ratio (IH) was calculated from the bulk and tapped densities to evaluate the powder’s flowability, using Equation (6). The angle of repose (α) and powder flow time (t″) were assessed using a 10 cm fixed-height funnel with a 1 cm orifice, in accordance with the European Pharmacopeia guidelines (2.9.36). A precise mass of powder was poured into a closed-end funnel mounted on a stand, allowing the powder to flow freely through the funnel’s orifice by gravity. The powder heap was assumed to form a right circular cone. The heap height (h) and the base diameter (2r) were measured directly with a ruler. The time taken for the powder to flow through the funnel, expressed in seconds per 10 g of powder, was recorded as t″. Additionally, the angle of repose was calculated based on the height (h) and radius (r) of the powder pile using Equation (7).

(6) $$\mathrm{HI}=\frac{D_{c}}{D_{a}}$$

(7) $${\alpha =\tan}^{-1}\left(\frac{h}{r}\right)$$

- Lubricity/Stability: The loss on drying (%HR) for approximately 1 g of powder was determined using a moisture analyzer (MX-50, A&D, Tokyo, Japan). An accurate initial mass (M_1_) was placed into an aluminum pan and heated at 105 ± 2 °C until a constant mass (M_2_) was achieved. The loss on drying was then calculated using Equation (8). Hygroscopicity (%H) was assessed by placing a pre-weighed 5 cm diameter polyethylene cup containing approximately 250 mg of powder (H_1_) into a sealed container maintained at 75 ± 5% relative humidity at ambient temperature. After 24 h, the cup was re-weighed (H_2_), and the percentage of weight gain was calculated using Equation (9) to determine the powder’s hygroscopicity.

(8) $$\%\mathrm{HR}=\frac{\left(M_{1}\;-\;M_{2}\right)}{M_{1}}\;\times \;100$$

(9) $$\%H=\frac{\left(H_{2}\;-\;H_{1}\right)}{H_{1}}\;\times \;100$$

- Lubricity/Dosage: The particle size fraction below 45 μm (%Pf) indicates the proportion of powder passing through a 45 μm sieve. This test was performed using a sieve vibrator (AS 200 Control, Retsch, Haan, Germany) at amplitude 1.0 g for 10 min with approximately 20 g of powder. The Homogeneity Index (Iθ) was assessed using the same sieve vibrator with four different sieve sizes (45, 106, 212, and 355 μm). The selection of sieve sizes has been justified by referencing the European Pharmacopeia and USP guidelines for particle size distribution analysis in pharmaceutical powders, which recommend these cut-offs to capture fine, medium, and coarse fractions relevant to direct compression performance. The Homogeneity Index was then calculated based on Equation (10). F_m_ represents the percentage of mass retained on a specific sieve, whereas $∆F_{\mathrm{mn}}$ denotes the difference in mass fraction between adjacent sieve sizes. A lower Iθ value indicates a uniform particle size distribution.

(10) $$I\theta =\frac{F_{m}}{100}+∆F_{\mathrm{mn}}$$

These radius values also facilitated the determination of Index Parameter; IP (Equation (11)), Parameter Profile Index; IPP (Equation (12)), and Index of Good Compressibility; IGB (Equation (13)).

(11) $$\mathrm{IP}=\frac{\mathrm{No}.P\;\geq \;5}{\mathrm{No}.\mathrm{Pt}}$$

where No.P ≥ 5 denotes the number of parameters with a value equal or higher than 5 while No.Pt refers to the total number of parameters studied.

IPP = Mean radius of all parameters(12)

(13) IGC=IPP × f

where f represents ratio of the polygon area to the circle area; therefore, to calculate the Good Compressibility Index (IGC), *f* is 0.952.

### 2.6. Estimation of the Co-Processed Ratio of Mannitol and Pregelatinized Rice Starch

The ratios of mannitol and PRS in the spray dried solution with ammonium bicarbonate were investigated according to the powder character data from SeDeM expert system. They were determined through mathematical evaluation to compensate for the deficiency factor of mannitol according to Equation (14) proposed by Suñé-Negre [16].

(14) $$\mathrm{CP}=100\;-\left(\frac{\mathrm{RE}\;-\;R}{\mathrm{RE}\;-\;\mathrm{RP}}\;\times 100\right)$$

where CP represents the percentage of mannitol in mixture solution. R, RE, and RP denote the mean-incidence radius value of the deficiency factor of co-processed excipients, mannitol, and PRS, respectively.

### 2.7. Morphology, Particle Size and Distribution of Spray Dried Co-Processed Mannitol/PRS

The morphological characteristics of the spray-dried co-processed mannitol/PRS were assessed using a scanning electron microscope (SEM). The samples were affixed to stubs with double-sided adhesive tape and subsequently coated with a thin layer of gold to enhance their visualization prior to analysis with a Philips XL 30 ESEM instrument (FEI Company, Hillsboro, OR, USA). The examination was performed using the acceleration voltage at 10–20 kV under low vacuum mode (0.7–0.8 Torr) with magnifications of 5000× and 10,000×.

The particle size and distribution were measured in triplicate using a laser diffraction instrument (Mastersizer 3000 Ultra, Malvern Panalytical, Worcestershire, UK) equipped with a dry powder dispersion unit (Aero S). The obscuration range was set between 0.1% and 3%, with a feed rate of 25% and an applied air pressure of 2 barg. An adequate amount of powder was loaded into the hopper with a gap of 2.5 mm. Data are presented as d10, d50, d90, and d (4,3), which indicate the particle sizes at which 10%, 50%, and 90% of the powder sample are smaller, as well as the volume-weighted mean diameter, respectively. The particle size distribution was reported in terms of span, as analyzed by the Mastersizer Xplorer software (version 5.20.2410.072).

### 2.8. Solid-State Characterization of Spray Dried Co-Processed Mannitol/PRS

The powders of mannitol, PRS, co-processed mannitol/PRS, and their physical mixture were analyzed to understand the physical properties of the co-processed excipient and ensure no chemical interaction occurred during spray drying.

- Differential scanning calorimetry (DSC)
  The DSC thermograms were obtained from a Differential scanning calorimeter (DSC-1, Mettler-toledo, Greifensee, Switzerland). To determine the thermo behavior, samples were analyzed at the temperature range of 25–250 °C with a heating rate of 10 °C/min. Physical mixture was blended with porcelain mortar with a selected ratio in accordance with ratio of co-processed mannitol/PRS. The temperatures of the characteristic transitions, i.e., onset (T_o_), peak (T_p_), end (T_e_), and peak area were recorded and finally the normalized enthalpy (ΔH) was calculated and displayed as J/g of sample weight.
- Fourier transform infrared (FTIR) spectroscopy
  The infrared spectra of samples were acquired using a Thermo Nicolet Nexus 470 FTIR spectrometer (Thermo Fisher, Waltham, MA, USA) with attenuated total reflectance (ATR) mode, covering the range of 4000–400 cm^−1^.
- X-ray powder diffraction (XRPD) analysis
  X-ray diffractograms of samples were obtained using a Miniflex II X-ray diffractometer (Rigaku, Tokyo, Japan) in reflection mode. The Bragg angle (θ) scanning region ranged from 10 to 60°, with a scan speed of 2.5° per minute.

### 2.9. Evaluation of Wettability

Approximately 250 mg of excipient powders were compressed into 11 mm diameter tablets using a hydraulic press (C, Carver, Hillsboro, OR, USA) at 9.81 kN. The tablets were stored in a desiccator before experiments. Contact angle measurements were performed using a sessile drop method with an Attention Theta Flow Optical Tensiometer (Biolin Scientific, Gothenburg, Sweden). A 6 µL glycerol drop was placed on the tablet surface via automated pipette, and images were captured every 0.02 s for 10 s. The contact angle was determined using OneAttension software Version 4.2.1 (r10106). Measurements were taken three times on different tablets of the same excipient and averaged. All experiments were conducted at ambient temperature (25 ± 2 °C) with 70 ± 5% relative humidity. To eliminate dissolution and swelling artifacts on tablet containing mannitol, glycerol (99% purity) which has negligible solubility toward mannitol was used instead of water. The liquid-solid affinity was calculated and represented as the work of adhesion (W_a_) between glycerol and the excipient on the tablet surface. It was determined using the surface energy of glycerol ($\gamma_{l}^{t}$), which is 64 mN/m, and the contact angle (θ) according to Equation (15).

(15) $$W_{a}=\;\gamma_{l}^{t}\left(1+\cos\theta \right)$$

The work of adhesion refers to the energy necessary to detach a glycerol droplet from the surface of an excipient. Consequently, a smaller contact angle signifies a greater work of adhesion, indicating increased interaction between the glycerol and the surface. This suggests that the excipient possesses enhanced wetting properties.

### 2.10. Mathematical Approach for Cetirizine Dihydrochloride Tablet Formulation

Initially, the target weight of the tablet utilizing flat-face tooling with a diameter of 6.3 mm was determined in accordance with the estimated fill depth of the tooling, as represented by Equation (16).

(16) Target weight=(Fill depth × Tip area) × Bulk density

where bulk density employed was that of the co-processed mannitol and PRS, rather than that of the complete formulation.

The ratios of the co-processed excipient and CET were derived using Equation (14), allowing for variations in the R value. Subsequently, the quantity of the co-processed excipient within the CET formulation was calculated, assuming a fixed quantity of CET at 10 mg. The amounts of the other excipients were determined based on concentration of 4%, 2%, and 1% *w*/*w* for sodium starch glycolate, sodium stearyl fumarate, and talc, respectively. Ultimately, the tablet weight corresponding to each value of R was estimated. The correlation between the estimated tablet weights and the respective R values was analyzed utilizing the Solver function in Excel. The optimal formulation was subsequently identified through the calculation of R values derived from the estimated tablet weights calculated using Equation (16).

### 2.11. Preparation of Cetirizine Dihydrochloride Immediate-Release Tablets Containing Co-Processed Mannitol and Pregelatinized Rice Starch

Directly compressible CET immediate-release tablets were formulated using co-processed mannitol and pregelatinized rice starch as diluents. Cetirizine dihydrochloride was uniformly blended with the co-processed excipient in a mathematically determined ratio, employing the geometric dilution method for 10 min. Subsequently, 4% *w*/*w* of sodium starch glycolate (SSG) was incorporated as a disintegrant and mixed for an additional 3 min. Finally, sodium stearyl fumarate (SSF) and talc, at concentrations of 2% and 1% *w*/*w*, respectively, were added as lubricants and mixed for 2 min. The powder blend was then compressed using an eccentric tableting machine (CMT 12, Charatchai, Bangkok, Thailand) equipped with 6.3 mm round, flat-face bevel edge tooling.

### 2.12. Comprehensive Evaluation of Physicochemical Properties of Cetirizine Dihydrochloride Tablets and Commercial Product

The uniformity of mass was determined following the procedure outlined in USP <905> uniformity of dosage units. Thirty tablets were sampled, and the 10 tablets were individually weighed. The standard deviation values are calculated, and the acceptance criterion was not more than 10%. According to USP <905>, the degree of uniformity in the amount of the CET in tablet required a content uniformity method. Ten randomly sampled tablets were individually weighed and dissolved in DI water to prepare a 50 μg/mL concentration. Each sample was then filtered through a 0.45 μm nylon syringe filter (Labfil, Shaoxing, China), and the amount of CET was analyzed in triplicate using a High-Performance Liquid Chromatography (HPLC) Agilent 1260 system equipped with Endura C18 (5 μm, 4.6 × 250 mm) column. The mobile phase consisted of 70% *v*/*v* methanolic solutions with a 0.5 mL/min flow rate. UV detection was performed at 230 nm. The linearity was established over the 5–200 μg/mL range with r^2^ = 0.9999. Subsequently, the acceptance value (AV) was calculated, with the maximum allowed acceptance value (L1) set at 15.0. The percentage of labeled amount was also calculated from those results and the acceptance limit ranged between 90 to 110.

Tablet thickness was manually measured using a digital outside micrometer (3109-24A, INSIZE, Siegen, Germany) and conducted on ten tablets. The crushing strength of these 10 tablets was determined using a tablet hardness tester (PTB-311 PharmaTest, Hainburg, Germany). Disintegration of CET immediate-release tablets was performed in accordance with the standard USP method. Six tablets were placed individually into each transparent tube of a basket-rack assembly. The disintegration apparatus was operated without a disk using distilled water at 37 ± 2 °C as medium. The time required for complete disintegration of each tablet was recorded.

### 2.13. Blending Homogeneity Using Raman Spectroscopy

To verify the homogeneity of co-processed excipient and low-dose drug in tablet, the Raman mapping was performed on the surface of a cetirizine tablet using a multi-ram spectrometer (Bruker Optic GmbH & Co. KG, Ettlingen, Germany). The laser source utilized was Nd:YAG with a wavelength of 1064 nm and a power of 450 mW. The InGaAs detector was employed, with each spectrum acquired at a resolution of 4 cm^−1^ over 16 scans within the range of 3000 to 100 cm^−1^. The mapping study covered an area of 3006 µm × 3280 µm, with a distance of 273.33 µm between each Raman spectrum. The characteristic peaks of co-processed excipient and cetirizine were identified using OPUS software (Bruker Optic GmbH & Co. KG, Ettlingen, Germany), and the peak areas were integrated to construct a 2D contour plot.

### 2.14. In Vitro Dissolution Studies

Dissolution studies of cetirizine (CET) in both the developed immediate-release tablet formulation and the reference product, Zyrtec^®^, were conducted utilizing the USP apparatus II (SR8PLUS Dissolution Test Station, Hanson Research, Chatsworth, CA, USA) set at a rotation speed of 50 revolutions per minute (rpm). According to the procedure in USP43, distilled water was specified as the dissolution medium (United States Pharmacopeial Convention, 2020). Each tablet was immersed in a vessel containing 900 mL of the medium, maintaining a temperature of 37 ± 0.5 °C. At predetermined time intervals, 5 mL aliquots of the dissolution medium were withdrawn and replaced with an equivalent volume of fresh medium. The collected samples were filtered through a 0.45 µm syringe membrane filter and subsequently analyzed using a UV–visible spectrophotometer at a wavelength of 231 nm. The percentage release of CET was calculated based on a linearity range of 3–23 µg/mL with an r^2^ value of 0.9996, and a graph plotting cumulative drug dissolution against time was generated. Each product was tested in triplicate.

The release profiles of CET from reference and test products were assessed using model-independent approaches based on the dissolution data. The differences (f_1_) and similarities (f_2_) of the profiles were calculated using the following equations:

(17) $$f_{1}\;=\;\frac{\sum_{t=1}^{n}\left|R_{t}-T_{t}\right|}{\sum_{t=1}^{n}R_{t}}\;\times \;100$$

(18) $$f_{2}=50\;\times \;\log\left\{{\left[1+\left(\frac{1}{n}\right)\sum_{t=1}^{n}{\left(R_{t}\;-\;T_{t}\right)}^{2}\right]}^{-0.5}\times \;100\right\}$$

where n represents the number of time points, while R_t_ and T_t_ denote the dissolution values of the reference and test products, respectively, at the time t. The dissolution profiles will be considered equivalence when f_1_ is less than 15 and f_2_ is greater than 50.

### 2.15. Statistical Analysis

The results are expressed as mean ± standard deviation (SD), with 95% confidence intervals applied. Statistical significance and curve fitting were evaluated using a two-tailed Student’s *t*-test, applying a *p*-value threshold of less than 0.05, and the Add-in Solver function, respectively. All statistical analyses were conducted using Microsoft Excel (Office 365, version 2403).

## 3. Results and Discussion

### 3.1. SeDeM-Based Characterization of Individual and Spray-Dried Powders

**Figure 1:**
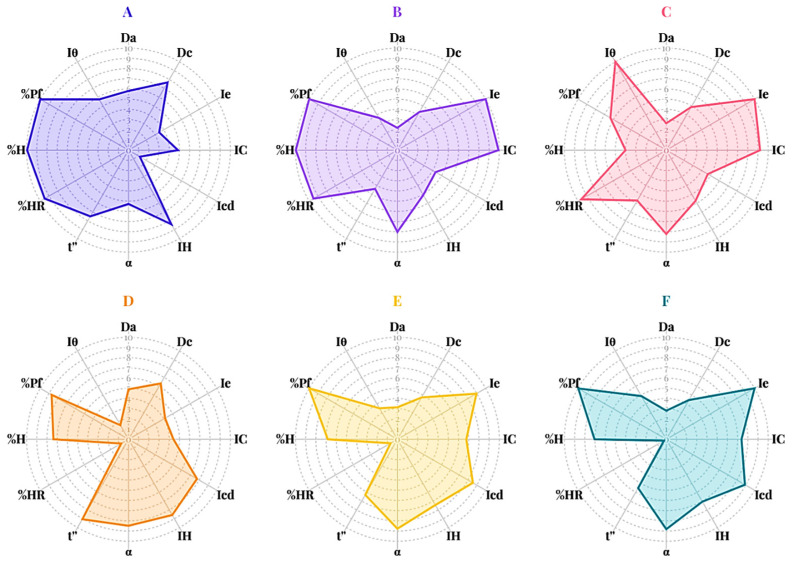
The SeDeM parameter diagrams of mannitol (**A**), spray-dried mannitol without ammonium bicarbonate (**B**), spray-dried mannitol with ammonium bicarbonate (**C**), pregelatinized rice starch; PRS (**D**), spray-dried PRS without ammonium bicarbonate (**E**), and spray-dried PRS with ammonium bicarbonate (**F**).

**Figure 2:**
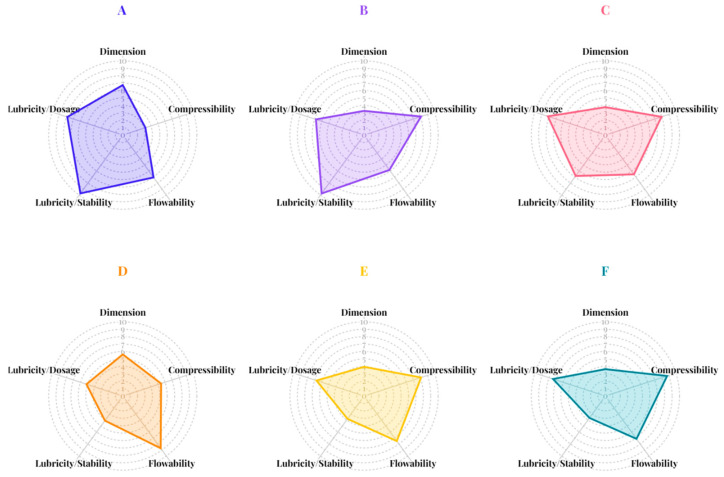
The SeDeM incidence factor diagrams of mannitol (**A**), spray-dried mannitol without ammonium bicarbonate (**B**), spray-dried mannitol with ammonium bicarbonate (**C**), pregelatinized rice starch; PRS (**D**), spray-dried PRS without ammonium bicarbonate (**E**), and spray-dried PRS with ammonium bicarbonate (**F**).

The powder characteristics of D-mannitol, pregelatinized rice starch (PRS) with determined degree of gelatinization of 85.85%, and spray-dried powders of mannitol and PRS—with or without ammonium bicarbonate—were assessed using the SeDeM expert system. The evaluation involved the twelve SeDeM parameters, followed by the calculation of radius values and incidence factors. The SeDeM diagrams representing these radii (Figure 1) and incidence factor values (Figure 2) were subsequently plotted.

Parameters falling below the established threshold of 5 were classified as deficient properties requiring attention. Both mannitol and PRS met the criteria for density parameters related to tablet dimensions, specifically bulk and tapped density radius values, i.e., D_a_ and D_c_. However, the two excipients demonstrated distinct performance profiles regarding compressibility. Mannitol exhibited all compressibility parameters (Ie, IC, and Icd) below acceptable values (Figure 1A), while PRS exceeded the acceptable limit only for its Icd value (Figure 1D). Although the low compressibility incidence factors of conventional mannitol suggest that higher compaction forces may be needed to achieve adequate tablet hardness, potentially limiting its suitability as a direct compression excipient, it was important to note that commercially available grades of mannitol—such as those produced via spray drying with ammonium bicarbonate—have been developed to enhance compressibility and tabletability. These modified grades exhibit improved performance, expanding the applicability of mannitol in direct compression formulations [8]. PRS exhibited higher compressibility due to its gelatinized structure, which increases plastic deformation under compression. These findings indicated superior compressibility properties of PRS compared to mannitol which was further supported by the compressibility incidence factor shown in Figure 2. Both excipients exhibited acceptable flowability properties when evaluated radius values and incidence factors. However, PRS exhibited a lubricity/stability incidence factor below the establish criteria due to reduced radius values of loss on drying (%HR). This observation suggested a high hygroscopic tendency, which may lead to moisture-induced degradation or instability in moisture-sensitive APIs. Although the lubricity/dosage incidence factor of PRS met the criteria, its board particle size distribution resulting in a homogeneity index (Iθ) below the acceptance limit. This deficiency raised concern regarding potential segregation during mixing, resulting in content uniformity issues during tablet manufacturing, particularly in low-dose formulations. In summary, compressibility emerged as the major limitation for mannitol whereas PRS presented challenges related to moisture sensitivity and potential segregation during manufacturing process.

The spray drying process, whether with or without ammonium bicarbonate, significantly influenced powders density characteristics. Both bulk and tapped densities decreased following spray drying, resulting in radius values (Figure 1C–F) and dimensional incidence factors (Figure 2C–F) that fell below acceptable thresholds. These findings suggested that tablets produced from spray-dried excipients would likely exhibit higher porosity and lower mechanical strength compared to those manufactured from conventional excipients when using the identical compression condition. SeDeM diagrams indicated that the spray drying, regardless of ammonium bicarbonate addition, enhanced the compressibility properties of both excipients which was a key parameter for direct compression methods. This improvement can be attributed to the increased surface area and greater porosity of the particles, which facilitate enhanced interparticle bonding, deforming, and rearrangement during compaction. Although the flowability parameters (IH and t″) slightly decreased, these values remained within acceptable limits. The addition of ammonium bicarbonate during spray drying increased the hygroscopicity of mannitol, which may be due to the creation of a porous structure as ammonium bicarbonate volatilizes [21]. This process resulted in high surface particles, enhancing water uptake capacity. Conversely, improvements in particle fraction (%Pf) and homogeneity index related to lubricity/dosage incidence factor were observed, indicating that co-spray-drying with ammonium bicarbonate reduced risk of segregation contributing to dose uniformity during tablet manufacturing.

The Index Parameter (IP), Parameter Profile Index (IPP), and Index of Good Compressibility (IGC) were summarized in Table 2. The IPP represents the average radius value across all 12 SeDeM parameters, reflecting overall powder suitability for direct compression, while the IGC quantifies the compressibility performance by combining IPP with a geometric factor to indicate the material’s ability to form robust tablets. All excipients, including those spray-dried with and without ammonium bicarbonate, exhibited IP values above 0.5 and IPP and IGC values above 5.00, confirming their suitability for direct compression. Notably, spray-dried mannitol demonstrated the highest IPP (7.34) and IGC (6.79), while mannitol and PRS co-spray-dried with ammonium bicarbonate also maintained IP, IPP, and IGC above the direct compression thresholds. Co-spray-drying with ammonium bicarbonate was selected not only for its ability to enhance porosity and reduce particle cohesion, which improves powder flow and tablet tensile strength, but also for its volatile nature, which leaves minimal residue after spray drying process. Although these co-processed excipients show excellent compressibility, further evaluation of segregation behavior is required.

Furthermore, a key limitation of the SeDeM approach is potential multicollinearity between Dimension and Compressibility incidence factors, since both derive from bulk and tapped density measurements. While the SeDeM system distinguishes these factors conceptually, Dimension reflects space-filling properties and Compressibility reflects densification under pressure. To improve predictive confidence for direct compression performance, future work should employ Variance Inflation Factor (VIF) analysis or Principal Component Analysis (PCA) to quantify and mitigate collinearity effects.

### 3.2. Mathematical Estimation of Mannitol and Pregelatinized Rice Starch Ratio

**Figure 3:**
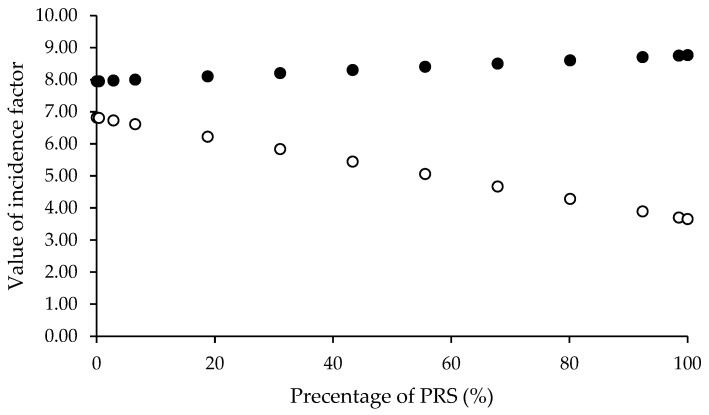
Relationship between percentage of pregelatinized rice starch (PRS) with compressibility incidence factor, R_C_, (●) and calculated lubricity/stability incidence factor, R_L_, (○).

For direct compression application, the compressibility incidence factor was identified as the critical parameter for optimizing the mannitol-to-PRS ratio. The proportions in the spray-dried solution were calculated using Equation (14), where CP represents the percentage of mannitol in the co-processed excipient. The variable R denotes the compressibility incidence factor values assigned to spray-dried mannitol (RE = 7.96), spray-dried PRS (RP = 8.76), and co-processed mannitol/PRS (R_C_). Any calculations resulting in negative mannitol percentages were excluded from consideration. The PRS percentages for each valid R_C_ value were calculated as the complement to 100%. Figure 3 illustrated the relationship between PRS percentage and R_C_ values, demonstrating that an increase in PRS content improved the compressibility of the co-processed excipient. To evaluate hygroscopicity tendencies, the same ratios were subsequently used to estimate the lubricity/stability incidence factor. The lubricity/stability incidence factor values for mannitol and PRS were 6.82 and 3.65, respectively, designated as variable RE and RP in Equation (14). The resulting R_L_ revealed that lubricity/stability incidence factor (R_L_), ranging from 3.65 to 6.82, exhibited an inverse relationship with PRS percentages (0.03 to 99.99%) implied that higher PRS content increased the hygroscopic tendency of the co-processed excipient. As PRS concentration increased, the lubricity/stability factor (R_L_) decreased. This trade-off must be balanced to avoid moisture-related degradation while maintaining compressibility. Considering an incidence factor threshold of 5, PRS content should not exceed 57.38% to maintain acceptable hygroscopicity stability. Based on recommended proportion levels for partial pregelatinized starch as a tablet binder, four R_L_ values (6.75, 6.56, 6.31, and 6.09) corresponding to PRS concentrations of 2%, 8%, 16%, and 23% *w*/*w* were selected for further investigation. These specific formulations were prepared to assess the SeDeM powder characteristics of the spray-dried co-processed mannitol/PRS system.

### 3.3. Evaluation of Spray Dried Co-Processed Mannitol/PRS

**Figure 4:**
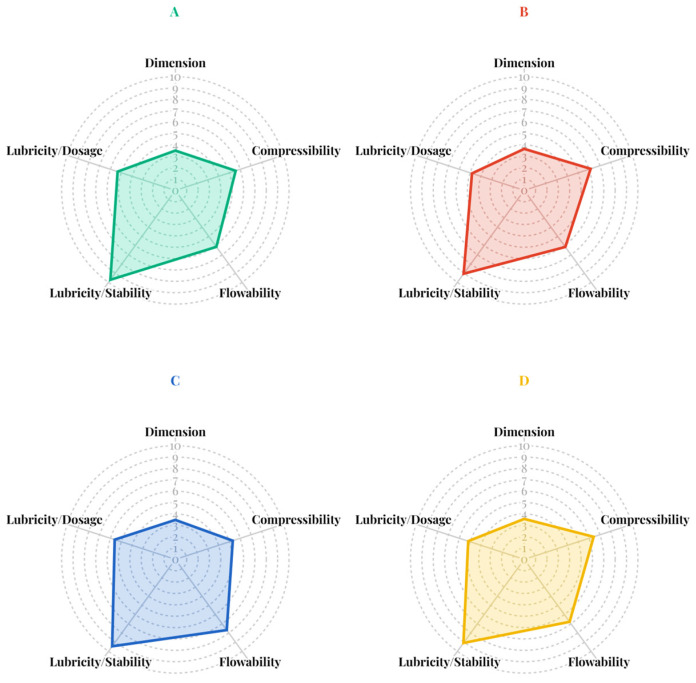
SeDeM incidence factor diagrams of co-processed spray-dried mannitol/PRS at R_L_ values of 6.75 (**A**), 6.56 (**B**), 6.31 (**C**), and 6.09 (**D**).

The SeDeM powder characteristics of the four co-processed mannitol/PRS (Figure 4) revealed that co-spray drying with ammonium bicarbonate effectively enhanced the lubricity/stability incidence factor while maintain favorable the compressibility and flowability. However, the dimensional incidence factor values for all formulations fell below the acceptable threshold. This was attributed to the formation of bulky, low-density particles resulting from the co-processing method. The co-processed mannitol/PRS formulation with R_L_ of 6.31, comprising 98% *w*/*w* mannitol and 2% *w*/*w* PRS, was selected for subsequent studies, as it exhibited superior lubricity/stability (9.45) along with acceptable incidence factor values for compressibility (5.31), flowability (7.69) and lubricity/dosage (5.61).

### 3.4. Morphology and Particle Size Analysis of Spray Dried Co-Processed Mannitol/PRS

**Figure 5:**
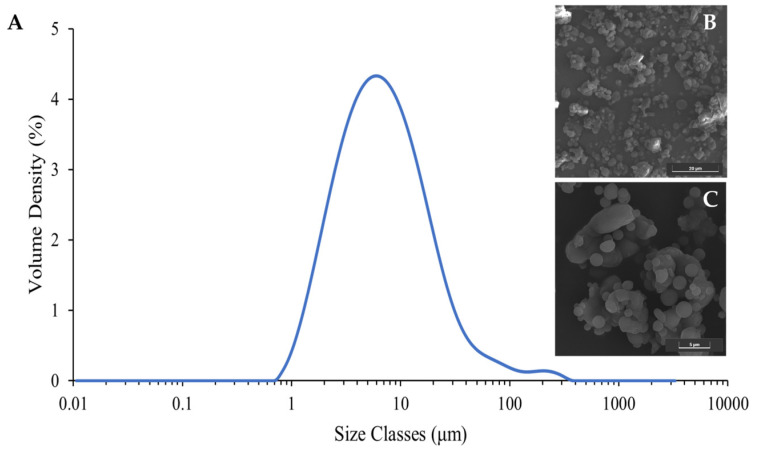
Particle size distribution (**A**) and surface morphology of spray dried co-process mannitol/PRS with magnifications of 5000× (**B**) and 10,000× (**C**).

According to the results from SeDeM approach, it was essential to evaluate the segregation tendency of the co-processed mannitol/PRS. To achieve this, particle morphology and size distribution were analyzed. SEM images (Figure 5B,C) revealed a mixture of irregular particles measuring between 10 and 15 μm and smooth, spherical particles ranging from 2 to 4 μm. These findings indicated the mannitol and PRS particles formed simultaneously during spray drying, leading to a close physical association without complete molecular fusion. Laser diffraction analysis expressed a monodisperse particle size distribution with a span value of 3.294 ± 0.025 (Figure 5A).

The particle size of the spray dried co-process excipient was described as monodispersed. Particle size analysis revealed d10, d50, and d90 values of 2.09 ± 0.00, 6.47 ± 0.03, and 23.4 ± 0.26 μm, respectively, and the calculated span was 1.64, demonstrating a relatively narrow size distribution. The volume-weighted mean diameter (d (4,3)) of the spray-dried co-processed mannitol/PRS was 12.7 ± 0.49 μm, consistent with the SEM observations. Although the presence of noticeable different particles, i.e., spherical versus irregular, may lead to segregation risk if density or surface energy notably varies. Considering the high air pressure condition applied during laser diffraction measurement, it could be presumed that these attached particles were likely capable of withstanding the mechanical stresses of the tablet blending process without significant segregation. Nonetheless, the homogeneity of the excipient distribution within tablets should be further investigated to ensure uniformity in the dosage form.

### 3.5. Solid-State and Surface Characterization of Spray Dried Co-Processed Mannitol/PRS

Co-processed excipients must not undergo chemical structure changes, therefore no interaction between mannitol and PRS should occur. To assess potential chemical interaction during spray drying, the co-processed mannitol/PRS with an R_L_ value of 6.56 was analyzed using DSC, FTIR and XRPD. Spectra of the individual components (mannitol and PRS) and their physical mixtures were compared with that of the co-processed product.

**Figure 6:**
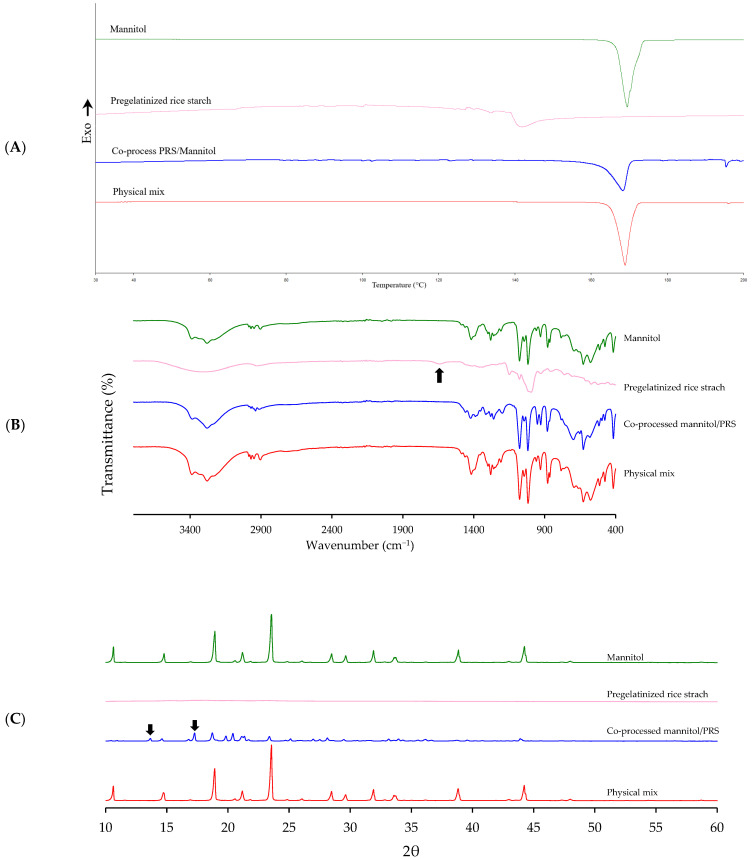
Solid-state characterization of mannitol, PRS, co-processed mannitol/PRS and physical mixture corresponding to R_L_ 6.56 by DSC (**A**), FTIR (**B**), and XRPD (**C**).

DSC thermogram (Figure 6A) revealed that the integrated enthalpy of the co-processed excipient (236.02 J/g) was lower than that of the physical mixture and mannitol (286.39 and 277.35 J/g). This suggested a possible, but weak, interaction between mannitol and PRS. The melting peak temperature of the co-processed mannitol/PRS (168.87 °C) was slightly lower than that of mannitol and physical mixture (169.5 and 168.93 °C, respectively), indicating slightly structural modifications during the spray-drying process. This modification was not substantial enough to imply significant chemical interaction. Minor shifts in melting temperature and enthalpy suggested physical interaction or microstructural changes, such as partial amorphization or hydrogen bonding, but not covalent chemical interaction. Moreover, the onset and end temperatures of the co-processed mannitol/PRS (163.36 °C and 170.11 °C, respectively) were also comparable to those of mannitol (166.73 °C and 171.70 °C, respectively) but significantly differed from PRS (138.98 °C and 147.45 °C, respectively). These findings implied that the thermal behavior of the co-processed excipient was predominantly influenced by mannitol. Overall, the DSC results confirmed that while minor physical interactions or microstructural changes may exist between mannitol and PRS, there was no evidence for significant chemical bonding or novel compound formation. FTIR spectra further confirmed the absence of chemical interaction as no additional peaks or shifts in characteristic functional bands were observed in the co-processed excipient compared to the individual components and their physical mixture. The O-H stretching vibrations (3700 to 3000 cm^−1^) and C-H stretching vibrations of -CH and -CH_2_ groups (3000 to 2800 cm^−1^) remained consistent. Specifically, mannitol peaks at 1421, 1260, 1077, and 627 cm^−1^, corresponding to C-H deformation in -CH_2_, O-H deformation in -CH_2_OH and -CHOH groups, and hydrogen deformation in OH structures, respectively [22], were preserved in both the co-processed and physical mixture spectra. The 1635 cm^−1^ band associated with C-O-C stretching in PRS (black arrow in Figure 6B), representing water molecules bound to starch [23], was absent in the spectra of both co-processed mannitol/PRS and physical mix. This absence was likely due to the relatively low PRS content (i.e., 2% *w*/*w*) in the co-processed excipient. Moreover, the absent of peak at 3102 cm^−1^ (Figure 6B), corresponding to NH_4_^+^ stretch of the ammonium bicarbonate molecule [24] indicated that no residual pore forming agent remained in the resulting powder.

XRPD analysis revealed that mannitol, co-processed mannitol/PRS, and physical mixture retained their crystalline characteristics, exhibiting an A-type crystalline pattern, whereas PRS displayed an amorphous structure. Mannitol showed characteristic peaks at 2θ of 10.64°, 14.78°, 23.56°, and 29.63°, which are typically indicative of the β-polymorph. The co-processed mannitol/PRS showed reduced peak intensities along with additional α-polymorph peaks, specifically at 13.66° and 17.27° (black arrows in Figure 6C). These changes were likely to be attributed to rearrangement in the crystal structure during the spray drying process [25,26,27]. The meta-stable form (α-polymorph) of spray-dried mannitol is influenced by droplet size and drying time. Under fast drying conditions, mannitol may not have sufficient time to crystallize into its stable polymorphic form (β-polymorph). Large droplets are expected to dry more slowly than small droplets, thereby retaining more moisture, which facilitates crystallization to the stable β-polymorph [28]. Therefore, the co-processed excipient retained the dominant β-mannitol crystalline structure while also exhibiting minor α-polymorph peaks, indicating partial polymorphic transformation without loss of overall crystallinity.

Consequently, the presence of PRS and ammonium bicarbonate in co-processed excipients may induce metastable mannitol formation by prolonging droplet drying time. This may affect the stability of the co-processed excipient and requires further investigation.

Collectively, the results from DSC, FT-IR, and XRPD confirmed that no significant chemical interaction occurred between mannitol and PRS during spray drying with ammonium bicarbonate. These findings provide insights into the physical and structural characteristics of the co-processed excipients, including crystalline transformations without evidence of molecular-level bonding.

In addition to physicochemical stability, evaluating surface properties such as wettability is essential to tablet performance predicting, particularly in terms of disintegration and dissolution behavior. The wettability of co-processed mannitol/PRS was summarized in Table 3. Contact angles below 90° all four excipients confirmed their intrinsically hydrophilic nature. No significant difference in contact angle (θ) at the 95% confidence interval (*p*-values of 0.107) suggested that the co-processing by spray drying did not alter the inherent wettability. Additionally, the co-processed mannitol/PRS exhibited the lowest W_a_, implied that spray drying process effectively increased surface hydrophobicity and reduced liquid-solid interfacial energy compared with PRS, physical mixing, and mannitol, respectively.

### 3.6. Formulation Design and Optimization of Cetirizine Dihydrochloride Immediate-Release Tablets

Co-processed mannitol/PRS with R_L_ 6.56 was employed as a filler in the development of cetirizine dihydrochloride (CET) immediate-release tablets. The optimal ratio between CET and co-processed excipient was determined using the application of Equation (13), wherein CP represents the percentage of co-processed mannitol/PRS in the blended powder. The variables R_F_, RE, and RP denote the mean-incidence radius value of the flowability factor of formulation, co-processed mannitol/PRS (5.31), and CET (5.29), respectively. Based on the systematic calculations using R_F_ values ranging from 5.0 to 7.6, the corresponding CP values fall within the range of 12.91% to 96.92%.

In pharmaceutical solid dosage form, the API content is fixed according to therapeutic requirement. In this case, each tablet was designed to contain 10 mg of CET. Additional excipients were incorporated at predetermined concentrations: 4% sodium starch glycolate (SSG), 1% sodium stearyl fumarate (SSF), and 1% talc. The quantity of co-processed mannitol/PRS was subsequently calculated based on CP values corresponding to each R_F_. The total tablet weight was computed as the sum of all formulation components: CET, co-processed mannitol/PRS, SSG, SSF, and talc.

**Figure 7:**
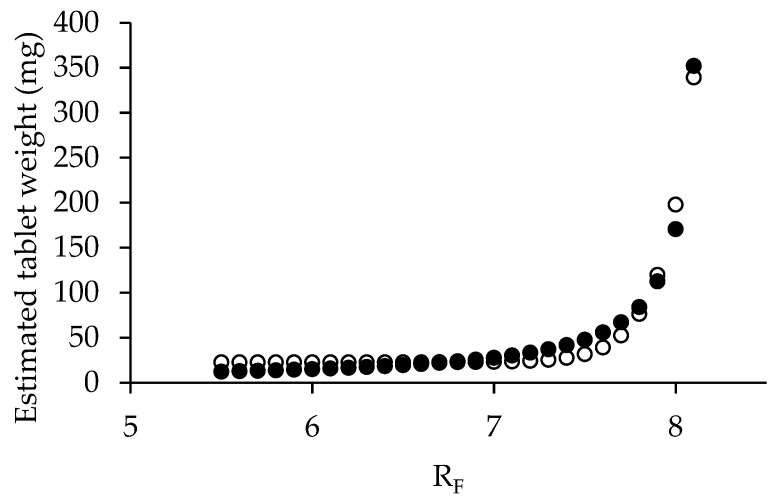
Exponential relationship between estimated tablet weight and R_F_ values (●) and fitting (○).

The relationship between the estimated tablet weight (y) and R_F_ (x) was analyzed using an exponential curve fitting with the Solver function in Microsoft Excel (Figure 7). The resulting natural exponential function demonstrated an excellent correlation with an r^2^ value of 0.9776. Final tablet weight calculations incorporated the bulk density of co-processed mannitol/PRS (R_L_ 6.56) as representative of the formulation’s bulk density. A target weight of 89.50 mg was established on the dimensions of the 6.3 mm flat-faced tooling, which included a fill depth of 10 mm, a cup volume of 0.3117 mL, and a tip area of 31.1725 mm^2^. Finally, the optimal R_F_ value was determined to be 7.3, corresponding to a formulation containing 87% co-processed mannitol/PRS. The complete formulation composition was subsequently detailed in Table 4.

### 3.7. Physicochemical and Homogeneity Evaluation of Cetirizine Dihydrochloride Immediate-Release Tablets

The developed CET tablets complied with pharmacopoeia specifications for both mass uniformity (84.80 ± 2.02 mg) and content uniformity, achieving an acceptance value of 9.26. These results indicated that the final powder blend possessed sufficient flowability to facilitate consistent die filling and uniform tablet weight distribution. The drug content was 91.16 ± 0.80% of labeled amount falling within acceptable limits. However, a notable observation was the loss of API during the tumbling process. Low content uniformity is a common issue, particularly when employing direct compression. Adhesion of API to the blender walls or other equipment surfaces may result in significant API loss and poor distribution within the blend. Additionally, over-blending can exacerbate demixing by promoting deagglomeration of API clusters. To mitigate these issues, an overage during scale-up manufacturing was recommended to compensate for potential API loss and ensure uniform drug distribution.

Physical characterization confirmed uniformity in tablet dimensions and mechanical strength, with thickness and tensile strength values of 2.005 ± 0.005 mm and 77.70 ± 12.09 N, respectively. The tablets also exhibited acceptable disintegration time within 1.85 ± 0.46 min. These results demonstrated that the optimized formulation produced tablets that met all predetermined physicochemical and quality criteria.

**Figure 8:**
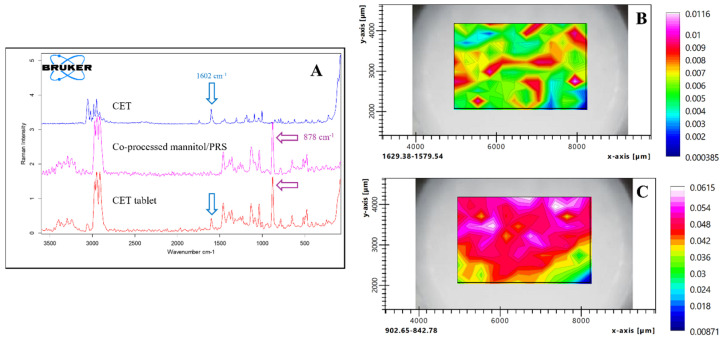
Raman spectra and chemical mapping of CET, co-processed mannitol/PRS and CET tablets (**A**) and spatial distribution of CET (**B**) and co-processed mannitol/PRS (**C**) in CET tablet where the white area indicates the high concentration level, and the blue area shows the low concentration level.

Raman chemical mapping was employed to assess the homogeneity of the CET and co-processed excipients across the tablet surface [29]. The representative peaks for those two compounds were identified at 1602 cm^−1^ and 878 cm^−1^, respectively. These peaks do not overlap with those of other excipients, allowing them to effectively represent each substance (Figure 8A). The integral areas of the characteristic peaks were utilized to generate the chemical mapping, with the contour colors on the map indicating the peak areas. The characteristic peaks of cetirizine were predominantly observed throughout the mapping area, primarily in green and red, signifying homogeneity within the tablet (Figure 8B). Co-processed mannitol/PRS, being the main component of the tablet, exhibited a larger peak area compared to the CET, which consists of 11.89% of the formulation. Figure 8C illustrated the distribution of co-processed mannitol/PRS across the mapping area. The distributions of cetirizine and co-processed mannitol/PRS were independent, reflecting the random arrangement of each tablet component within the mapping area.

**Figure 9:**
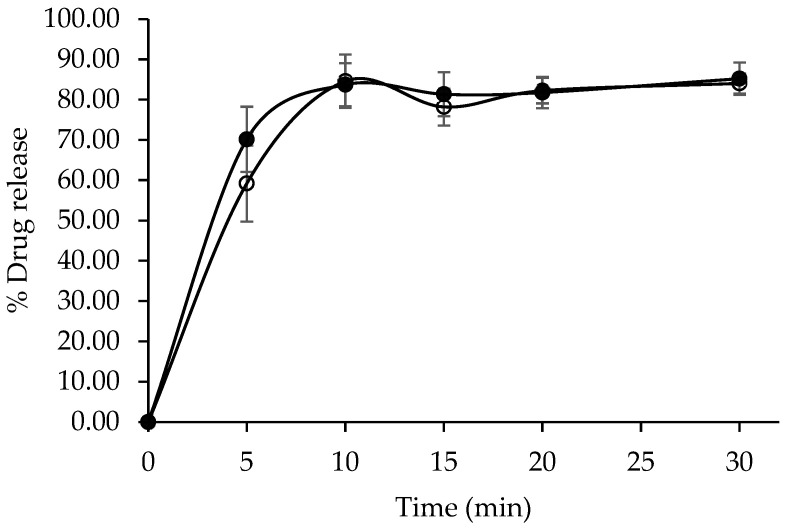
In vitro dissolution profiles of developed CET immediate-release table (●) and Zyrtec^®^ (○).

The dissolution profiles of the developed CET tablets and the commercial reference product (Zyrtec^®^) were evaluated in water as the dissolution medium. Both formulations exhibited characteristic immediate-release behavior, with drug release exceeding 80% within 10 min (Figure 9). A statistical comparison of the dissolution profiles using model-independent approaches resulted in a dissimilarity factor (f_1_) of 4.28 and a similarity factor (f_2_) of 64.03. These values fall within the accepted regulatory criteria (f_1_ < 15 and f_2_ > 50), demonstrating that the developed formulation was bioequivalent to the reference product with respect to in vitro dissolution performance.

## 4. Conclusions

This study successfully developed and optimized a novel co-processed excipient composed of mannitol and pregelatinized rice starch (PRS) for direct compression application. Utilizing the SeDeM expert system, the individual limitations of mannitol (poor compressibility) and PRS (moisture sensitivity and segregation risk) were systematically identified. The incorporation of ammonium bicarbonate during spray drying was particularly effective in enhancing compressibility, reducing segregation potential, and maintaining acceptable flowability of co-processed mannitol/PRS. Mathematical modeling based on SeDeM incidence factors enabled precise optimization of the mannitol-to-PRS ratio, with the formulation containing 87% co-processed excipient (R_L_ 6.56) selected for tablet development. Solid-state analyses (DSC, FTIR, and XRPD) confirmed the absence of chemical interactions between mannitol and PRS. When applied in the formulation of immediate-release cetirizine dihydrochloride (CET) tablets, the optimized co-processed excipient resulted in tablets with uniform mass, acceptable hardness, rapid disintegration (less than 2 min), and over 80% drug release within 10 min—demonstrating bioequivalence to the commercial reference product (Zyrtec^®^). Raman mapping further confirmed the homogeneous distribution of both the API and excipients within the tablet matrix. Collectively, these findings validate the effectiveness of the co-processed mannitol/PRS excipient for direct compression and its suitability for immediate-release formulations. This work also highlights the utility of the SeDeM expert system as a rational, predictive tool for excipient development, facilitating efficient formulation design while reducing reliance on empirical trial-and-error methods. The proposed approach offers a robust platform for the scalable production of high-quality oral solid dosage forms.

## Figures and Tables

**Table 1:** Parameters and their conversion equations for the SeDeM expert system.

Incidence Factor	Parameter	Symbol	Units	Conversion Equation *
Dimension	Bulk density	D_a_	g/mL	10v
	Tapped density	D_c_	g/mL	10v
Compressibility	Inter-particle porosity	Ie	-	$\frac{10v}{1.2}$
	Carr Index	IC	%	$\frac{v}{5}$
	Cohesion Index	Icd	N	$\frac{v}{200}$
Flowability	Hausner ratio	IH	-	$\frac{(30-10v)}{2}$
	Angle of repose	α	°	$10-\frac{v}{5}$
	Powder flow	t″	s	$10-\frac{v}{2}$
Lubricity/Stability	Loss on drying	%HR	%	10−v
	Hygroscopicity	%H	%	$10-\frac{v}{2}$
Lubricity/Dosage	Particle size < 45 μm	%Pf	%	$10-\frac{v}{5}$
	Homogeneity index	Iθ	-	500v

* The value of v in the conversion equation indicates the experimentally or mathematically determined value of the corresponding SeDeM parameter.

**Table 2:** The Index Parameter (IP), Parameter Profile Index (IPP), and Index of Good Compressibility (IGC) of excipients.

Sample	IP	IPP	IGC
Mannitol	0.75	6.87	6.35
Spray dried mannitol	0.75	7.34	6.79
Spray dried mannitol with ammonium bicarbonate	0.67	6.65	6.15
Pregelatinized rice starch (PRS)	0.75	6.38	5.90
Spray dried PRS	0.75	6.54	6.05
Spray dried PRS with ammonium bicarbonate	0.67	6.37	5.90

**Table 3:** Surface Wettability Properties of Mannitol, PRS, Physical Mixture, and Co-Processed Formulations.

Excipients	Contact Angle, θ (°)	Work of Adhesion, W_a_ (mJ/m^2^)
Mannitol	42.06 ± 1.32	111.51 ± 0.99 *
Pregelatinized rice starch (PRS)	48.22 ± 2.28	106.62 ± 1.88
Physical mixture	47.48 ± 3.28	107.79 ± 1.34
Co-processed mannitol/PRS	48.11 ± 4.41	104.74 ± 3.90 *

* *p*-value = 0.0297 at 95% confidence interval.

**Table 4:** Optimized composition of cetirizine dihydrochloride immediate-release tablets.

Composition	mg per Tablet	%*w*/*w*
Cetirizine dihydrochloride	10.00	11.90
Co-processed mannitol/PRS	68.49	80.95
Sodium starch glycolate	3.14	3.57
Sodium stearyl fumarate	1.63	2.38
Talc	0.82	1.19
Total	84.07	100.00

## Data Availability

Data is contained within the article.

## Abbreviations

The following abbreviations are used in this manuscript:

API	Active Pharmaceutical Ingredient
AV	Acceptance Value
CET	Cetirizine Dihydrochloride
CP	Percentage of co-processed excipient in mixture solution
DSC	Differential Scanning Calorimetry
f_1_	Dissimilarity factor
f_2_	Similarity factor
FTIR	Fourier-Transform Infrared Spectroscopy
HPLC	High-Performance Liquid Chromatography
HI	Hausner ratio
Ie	Inter-particle porosity
IC	Carr Index
Icd	Cohesion Index
IGB	Good Compressibility Index
IGC	Index of Good Compressibility
IP	Index Parameter
IPP	Parameter Profile Index
Iθ	Homogeneity Index
PRS	Pregelatinized rice starch
PXRD/XRPD	X-Ray Powder Diffraction
SSG	Sodium Starch Glycolate
SSF	Sodium Stearyl Fumarate
t″	Powder flow time
USP	United States Pharmacopeia
Wa	Work of adhesion
%HR	Loss on drying
%H	Hygroscopicity
%Pf	Percentage of particles < 45 µm
